# Dynamic Cervical Myelopathy Misleading on Neutral Imaging: The Role of Flexion–Extension MRI

**DOI:** 10.3390/jcm15041333

**Published:** 2026-02-08

**Authors:** Leonardo Anselmi, Donato Creatura, Mario De Robertis, Ali Baram, Emanuele Stucchi, Gabriele Capo, Jad El Choueiri, Federico Pessina, Maurizio Fornari, Carlo Brembilla

**Affiliations:** 1 Department of Biomedical Sciences, Humanitas University, Via Rita Levi Montalcini 4, Pieve Emanuele, 20090 Milan, Italy; donato.creatura@humanitas.it (D.C.); mario.derobertis@humanitas.it (M.D.R.); emanuele.stucchi@humanitas.it (E.S.); jad.elchoueiri@st.hunimed.eu (J.E.C.); federico.pessina@hunimed.eu (F.P.); 2 Department of Neurosurgery, IRCSS Humanitas Research Hospital, Via Manzoni 56, Rozzano, 20089 Milan, Italy; ali.baram@humanitas.it (A.B.); gabriele.capo@humanitas.it (G.C.); maurizio.fornari@humanitas.it (M.F.); carlo.brembilla@humanitas.it (C.B.)

**Keywords:** dynamic cervical myelopathy, flexion–extension MRI, cervical myelomalacia, osteophyte, Hirayama disease, anterior cervical discectomy and fusion (ACDF)

## Abstract

**Background/Objectives:** Degenerative cervical myelopathy (DCM) may result from posture-dependent spinal cord compromise not detectable on neutral imaging. Dynamic MRI can uncover clinically relevant mechanisms underlying otherwise unexplained myelopathy and guide management. This report illustrates a dynamic cervical myelopathy phenotype revealed by flexion–extension imaging and its impact on surgical decision-making. **Methods:** A 49-year-old man presented with progressive bilateral upper-limb paresthesias, intrinsic hand atrophy, and distal weakness. Neutral cervical MRI, standard radiographs, and flexion–extension MRI were performed to investigate a suspected dynamic etiology, including differentiation from Hirayama disease. Surgical treatment consisted of anterior cervical discectomy and fusion (ACDF), with clinical and radiological follow-up. **Results:** Neutral MRI showed intramedullary T2 hyperintensity from C4 to C6 without static canal stenosis or frank compression, while radiographs demonstrated segmental kyphosis without instability. Flexion MRI revealed reproducible spinal cord contact with a small cranially located osteophyte at C5–C6, concordant with the myelopathic signal. ACDF at C4–C6 led to clinical improvement. One year later, recurrent symptoms from adjacent-segment pathology (C3–C4 myelopathic signal and C6–C7 foraminal disc herniation) required a second ACDF, resulting in durable neurological stability. **Conclusions:** This case demonstrates flexion-dependent cord–osteophyte conflict causing cervical myelomalacia in the absence of static stenosis. Dynamic MRI resolved a clinical–radiological mismatch and directly informed surgical planning. Recognition of dynamic myelopathy phenotypes and vigilance for adjacent-segment disease after fusion are essential for optimizing outcomes.

## 1. Introduction

Degenerative cervical myelopathy (DCM) is the most common cause of non-traumatic spinal cord dysfunction in adults and arises from a combination of static and dynamic mechanical factors that produce chronic injury [1,2,3]. Static factors—such as disc degeneration, osteophyte formation, ligamentum flavum hypertrophy, and OPLL—progressively narrow the cervical canal, creating chronic cord compression that induces secondary ischemic and inflammatory changes leading to demyelination and axonal injury [1,4].

Beyond static compression, dynamic factors related to cervical motion are important contributors to disease severity and progression. Cervical movement, particularly extension, can induce longitudinal deformation, shear stress, and microvascular compromise of the spinal cord, producing biomechanical stress that may not appear on neutral MRI [5]. These stresses help explain why some patients develop marked neurological deficits despite only modest or absent canal narrowing on static imaging [4,5].

Dynamic cervical myelopathy can also occur in younger adults without advanced spondylosis. Physiological extension alone can produce clinically significant cord compression even in the presence of minimal degenerative changes, highlighting the limitations of neutral MRI in detecting true functional stenosis [6,7]. Cervical sagittal parameters, including lordosis and segmental alignment, further modulate dynamic canal behavior and may influence both disease progression and postoperative outcomes [8].

Although neutral MRI remains the standard diagnostic tool, it often underestimates posture-dependent cord compromise. Early kinematic MRI studies demonstrated that cervical extension can significantly accentuate canal narrowing and spinal cord indentation, and subsequent dynamic MRI demonstrated higher-grade stenosis and T2 abnormalities detectable only in extension [9,10]. More recent work has confirmed that dynamic MRI can uncover additional levels of clinically relevant compression and provide information that meaningfully guides surgical decision-making [11,12,13].

Together, these findings support the concept of dynamic myelopathy, in which mechanical stress during physiological motion significantly contributes to spinal cord injury, and where dynamic MRI may provide essential diagnostic information not obtainable from static imaging alone.

## 2. Case Report

### 2.1. Clinical Background and Patient Information

A 49-year-old man presented with several months of progressively worsening bilateral upper-limb paresthesias, predominantly involving the hands, associated with visible intrinsic hand muscle atrophy and declining manual dexterity. He reported increasing difficulty with fine motor tasks during daily activities. He denied lower-limb weakness, gait disturbance at presentation, sphincter dysfunction, neck pain, or radicular symptoms. There was no history of trauma, inflammatory disease, or previous cervical spine surgery.

Neurological examination revealed distal upper-limb weakness, most pronounced in the intrinsic hand muscles, associated with sensory disturbance. No lower-limb involvement, or sphincter abnormalities were detected at that stage.

### 2.2. Diagnostic Assessment

**Figure 1:**
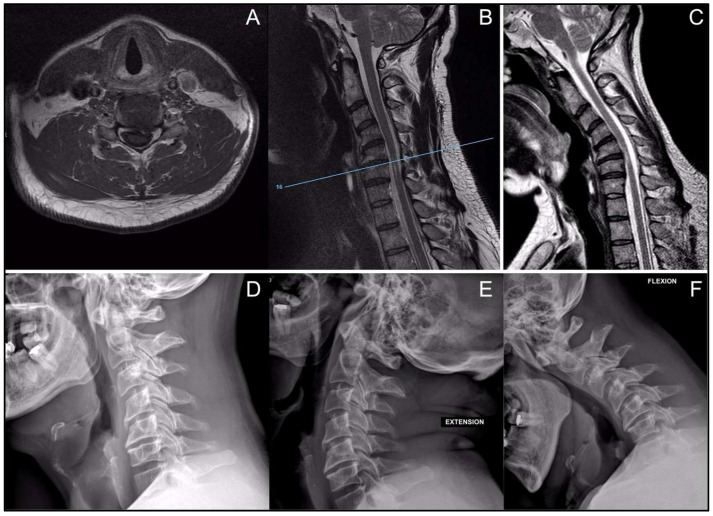
(**A**) Axial T1-weighted MRI at C5–C6 showing a small left-sided osteophyte. (**B**) Sagittal T2-weighted MRI demonstrating intramedullary hyperintensity (myelomalacia); axial level reference marker shown. (**C**) Dynamic sagittal T2-weighted MRI in maximal flexion demonstrating anterior cord–osteophyte contact at C5–C6; the T2 hyperintensity is located more caudally on neutral imaging, with flexion revealing the conflict. (**D**–**F**) Lateral cervical radiographs: neutral (**D**), extension (**E**), and flexion (**F**).

Neutral cervical MRI demonstrated distinct intramedullary T2 hyperintensity at the C4–C5 and C5–C6 levels, consistent with myelopathic signal change, without evidence of static canal stenosis, disc herniation, ligamentous hypertrophy, or other spondylotic elements capable of producing fixed spinal cord compression (Figure 1A,B). Given the absence of a clear compressive etiology on standard imaging, the patient was referred to the neurology service for further evaluation.

Following multidisciplinary discussion, cervical dynamic radiographs were obtained and demonstrated segmental cervical kyphosis on sagittal projection without translational or angular instability on flexion–extension views (Figure 1D–F). Comprehensive neurophysiological testing was performed to exclude primary neurological or motor neuron disease, and the results supported a cervical origin of the symptoms.

In the setting of a clinical–radiological mismatch with neurologic etiologies excluded, dynamic cervical MRI was obtained due to suspicion of Hirayama disease. Flexion imaging revealed anterior spinal cord contact against the posterior aspect of the C5–C6 disc space at the site of a small cranially located osteophyte, producing a reproducible indentation not present in neutral alignment and topographically concordant with the intramedullary T2 hyperintensity, supporting a posture-dependent mechanical mechanism of spinal cord injury (Figure 1C).

### 2.3. Surgical Intervention

Given the evidence of flexion-induced spinal cord compression and the progressive neurological deficits, surgical treatment was recommended to eliminate recurrent dynamic mechanical insult to the spinal cord. Although the most evident cord–osteophyte conflict was observed at C5–C6, flexion–extension MRI demonstrated increased segmental motion involving both C4–C5 and C5–C6, suggesting a broader hypermobile segment contributing to dynamic cord compromise.

**Figure 2:**
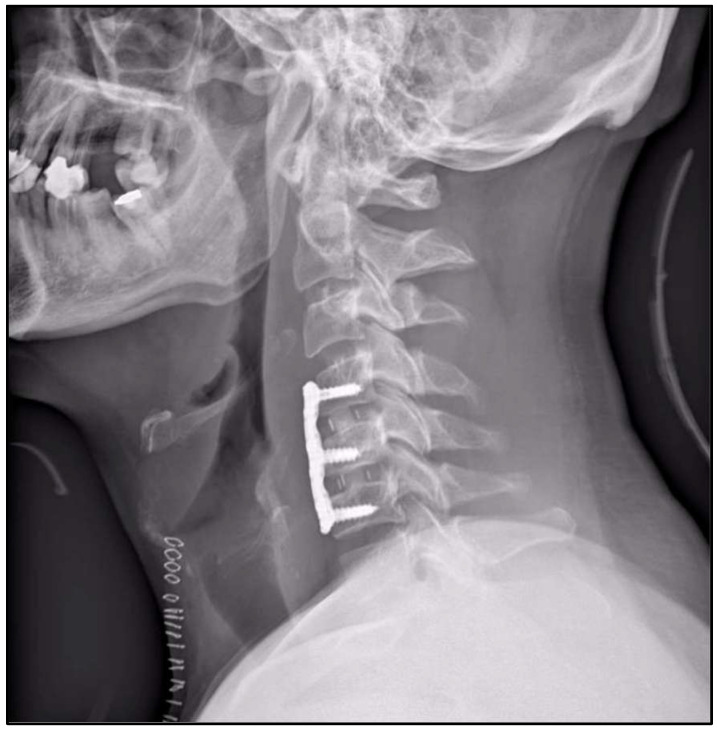
Postoperative lateral cervical radiograph demonstrating ACDF at C4–C5 and C5–C6 with interbody cages and an anterior plate spanning C4–C6.

Based on these findings, a two-level anterior cervical discectomy and fusion (ACDF) from C4 to C6 was selected to achieve adequate decompression and stabilization while minimizing the risk of persistent or recurrent flexion-related cord injury. The patient underwent ACDF at C4–C5 and C5–C6 using interbody cages and anterior plate fixation from C4 to C6 (Figure 2). The postoperative course was uneventful.

### 2.4. Follow-Up and Outcomes

Postoperatively, the patient experienced progressive neurological improvement, with recovery of hand strength and reduction in paresthesias, allowing return to full daily activities. Clinical and radiological follow-up demonstrated construct stability and sustained neurological improvement for approximately one year.

Approximately one year after the index procedure, the patient developed new symptoms, including right upper-limb radicular pain, progressive gait unsteadiness, and intermittent vertigo. Neurological examination revealed mild global weakness of the right upper limb, sensory reduction along the medial forearm and hand, and an unsteady Romberg test with medium-amplitude oscillations without collapse. Lower-limb reflexes were hypoactive, plantar responses remained flexor, and Hoffmann’s sign was negative.

**Figure 3:**
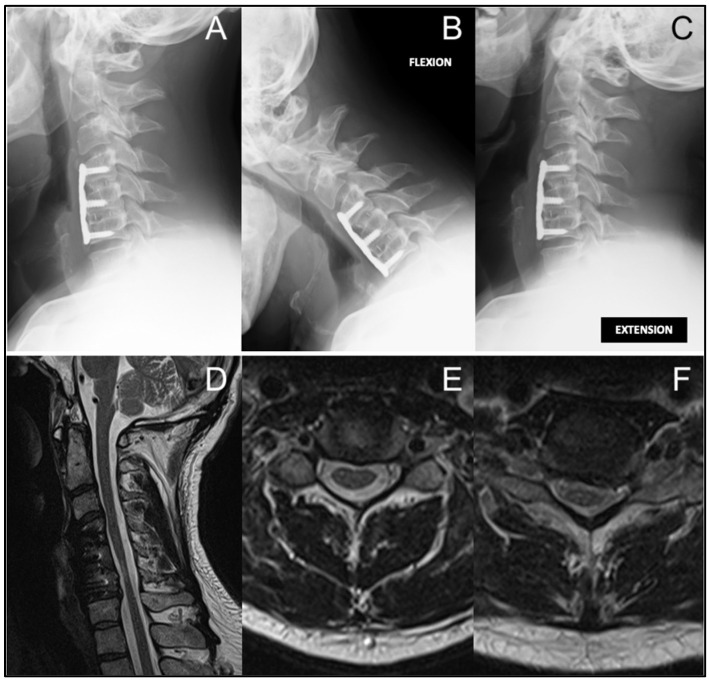
(**A**–**C**) One-year follow-up lateral cervical radiographs ((**A**) neutral, (**B**) flexion, (**C**) extension) showing intact anterior construct without displacement or segmental instability at C3–C4. (**D**–**F**) One-year follow-up cervical T2-weighted MRI ((**D**) sagittal, (**E**,**F**) axial) showing intramedullary T2 hyperintensity at C3–C4 (**E**), and a new left C6–C7 foraminal disc herniation compressing the C7 nerve root (**F**).

A new static MRI demonstrated postoperative stability at C4–C6, a new left-sided C6–C7 foraminal disc herniation compressing the C7 nerve root, and new intramedullary T2 hyperintensity at C3–C4, consistent with recurrent dynamic myelopathic involvement at an adjacent level (Figure 3). These findings correlated with clinical deterioration and indicated the need for revision surgery.

**Figure 4:**
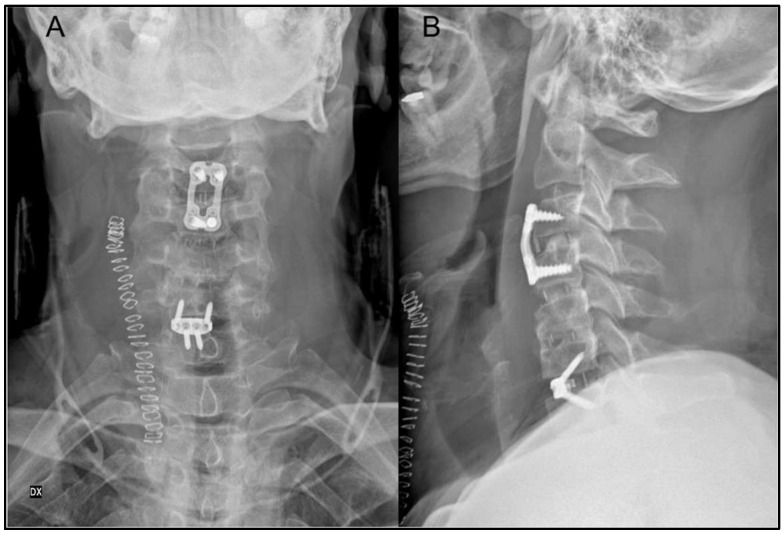
(**A**,**B**) Postoperative cervical radiographs (coronal (**A**), lateral (**B**)) showing ACDF at C3–C4 and C6–C7. The prior anterior plate from the C4–C6 construct was removed (solid fusion) to facilitate the new procedures. At C3–C4, a cage plus anterior plate was used due to intraoperative hypermobility; at C6–C7, a screw-anchored standalone cage was selected given lower segmental mobility.

Approximately 18 months after the index procedure, a second operation was performed. ACDF at C3–C4 was carried out using an interbody cage and anterior plate to restore stability at the most unstable segment. At C6–C7, an interbody device with integrated screws was selected due to relative segmental ankylosis and preserved structural stability (Figure 4). The prior C4–C6 anterior plate was removed following confirmation of solid fusion, also to minimize the risk of postoperative dysphagia. No intraoperative or immediate postoperative complications occurred.

Postoperatively, the patient experienced progressive clinical improvement. Radicular pain resolved early, gait normalized over subsequent weeks, and no new neurological deficits emerged. At long-term follow-up, approximately 10 years after the second procedure, the patient remains neurologically stable with sustained functional recovery.

### 2.5. Clinical Implications

This case highlights the importance of considering dynamic cervical myelopathy in patients presenting with intramedullary T2 hyperintensity in the absence of clear static compression on neutral MRI. Dynamic flexion MRI may reveal posture-dependent spinal cord conflict, resolve clinical–radiological mismatch, and directly influence surgical decision-making. Additionally, the occurrence of subsequent adjacent-level pathology underscores the potential multisegmental nature of dynamic cervical disease and the need for long-term clinical and radiological surveillance.

## 3. Discussion

The concept that cervical myelopathy arises from the interplay of static and dynamic injury mechanisms is well established. Extensive contemporary reviews describe how degenerative cervical myelopathy (DCM) results from fixed stenosis, sagittal malalignment, and repetitive motion-related stress that induces ischemia, inflammation, demyelination, and axonal degeneration [1,4]. Henderson et al. expanded this framework with the paradigm of stretch-associated injury, demonstrating that cervical motion, especially flexion/extension in the setting of kyphosis or subtle deformity, subjects the cord to longitudinal strain, shear forces, and microvascular insufficiency that may not be apparent on neutral MRI [5]. This model helps explain why some patients with minimal canal compromise on standard imaging display clinically significant myelopathy.

Dynamic factors can be clinically relevant even in the absence of advanced spondylosis. Physiological motion alone has been shown to generate meaningful cord compression in younger individuals with only mild degenerative changes, highlighting the presence of functional stenosis that may not be apparent on neutral MRI [6,7].

The importance of evaluating cervical pathology under physiological motion was demonstrated early by Muhle et al., who showed that canal narrowing and cord compression are often most pronounced in extension and may be underestimated or entirely absent in the neutral position [9]. Subsequent prospective studies by Zhang et al. and others confirmed that high-grade stenosis and intramedullary T2 hyperintensity can be accentuated—or even exclusively revealed—on dynamic imaging [10,13,14]. Additional work showed that incorporating dynamic MRI increases the number of detected compressive levels and improves alignment between clinical presentation and imaging findings [11].

More recent investigations further support integrating dynamic MRI into routine assessment, demonstrating that dynamic imaging frequently uncovers occult or underestimated stenosis and can meaningfully influence surgical planning [15]. Similarly, Makhchoune et al. reported that dynamic MRI not only identifies the severity and location of compression more accurately but also alters the recommended operative strategy by revealing pathology not seen on neutral studies [16]. These findings are consistent with the observations of Mahdavi et al., who described flexion–extension MRI as an evolving tool for guiding the diagnosis and management of DCM [17,18]. Large contemporary cohorts have also shown that extension systematically worsens stenosis, increases cord occupation, correlates with T2 signal changes, and predicts postoperative outcomes; multicenter analyses further underline the superior diagnostic reliability of dynamic over static MRI [13,18,19]. Together, these studies highlight the critical importance of evaluating motion-induced pathophysiology.

Beyond macroscopic compression, dynamic MRI can also reveal early microstructural injury. Dynamic diffusion tensor imaging (DTI) has demonstrated posture-dependent reductions in fractional anisotropy and increases in apparent diffusion coefficient at affected segments, findings consistent with acute axonal stress [20]. Multicenter prospective data further showed that dynamic DTI biomarkers distinguish myelopathic from non-myelopathic segments even when standard T2-weighted imaging appears normal [21]. These results suggest that dynamic imaging captures physiologically relevant spinal cord stress that static MRI is unable to detect.

Our case underscores these points. Despite marked intramedullary T2 hyperintensity at C4–C5 and C5–C6, neutral imaging showed no static compression. Dynamic flexion MRI revealed reproducible anterior cord impingement at C5–C6 against a small cranial osteophyte, resolving the clinical–radiological mismatch and supporting posture-dependent mechanical injury in the context of segmental kyphosis. The segmental distribution of the T2 hyperintensity, involving the same levels responsible for distal hand innervation, was concordant with the patient’s intrinsic hand muscle atrophy and weakness. This scenario also illustrates the practical differential with Hirayama disease: whereas this condition typically affects adolescents and young adults and represents a self-limiting, dural-based flexion myelopathy with a lower motor neuron–predominant phenotype, our middle-aged patient demonstrated a focal bony cord–osteophyte conflict without dural displacement or venous plexus enlargement, which may help explain a degenerative dynamic myelopathy [6,7].

In clinical practice, dynamic MRI should be reserved for selected patients, given cost considerations and the lack of standardized selection criteria. It is most useful in cases with clinical–radiological mismatch on neutral imaging, where it can clarify posture-dependent cord compromise.

The treatment of this still-evolving entity remains debated, without a universally accepted standard. Management in our case focused on eliminating the flexion conflict. In the literature, anterior approaches—or combined anterior–posterior strategies—are frequently employed to achieve this objective [22,23,24]. The subsequent adjacent-segment pathology requiring additional ACDF aligns with the known risk of adjacent segment disease after cervical fusion; however, it may also reflect the progression of an underlying multilevel dynamic condition rather than a purely fusion-related phenomenon, highlighting the need for surveillance and careful level selection in dynamic DCM [25]. Dynamic imaging was instrumental both in the initial diagnosis and in identifying subsequent symptomatic segments, thereby directly informing surgical planning.

In both operations, although intraoperative neurophysiological monitoring (IONM) is often considered the standard of care in surgery for myelopathy [26,27], its use was individualized in this case based on case-specific and institutional practice considerations. Specifically, the pathophysiology involved repetitive flexion-related mechanical conflict rather than fixed compression; adequate cord space was present intraoperatively, and no intraoperative or postoperative adverse neurological events occurred.

## 4. Conclusions

Dynamic cervical myelopathy should be considered in patients with myelopathic signs and intramedullary T2 abnormalities but no clear static compression. Dynamic MRI can unmask posture-dependent cord conflict, reconcile clinical–imaging discrepancies, and guide operative strategy. Distinguishing degenerative flexion conflict mechanisms from Hirayama disease is essential, and vigilance for adjacent-segment pathology after fusion is warranted. The emerging literature further supports dynamic imaging in suspected Hirayama phenotypes and refines diagnostic and therapeutic pathways [28,29].

## Data Availability

The original contributions presented in this study are included in the article material. Further inquiries can be directed to the corresponding author.

## Abbreviations

The following abbreviations are used in this manuscript:

ACDF	Anterior Cervical Discectomy and Fusion
DCM	Degenerative Cervical Myelopathy
DTI	Diffusion Tensor Imaging
IONM	Intraoperative Neurophysiological Monitoring
MRI	Magnetic Resonance Imaging
OPLL	Ossification of the Posterior Longitudinal Ligament
